# Supplementary material for: The Photorhabdus asymbiotica virulence cassettes deliver protein effectors directly into target eukaryotic cells
Source: eLife. 2019 Sep 17;8:e46259. doi: 10.7554/eLife.46259 (PMC6748792; doi:10.7554/eLife.46259)
Supplement: Supplementary file 1. — Oligonuclotides used for the construction of pAGAG based reporter plasmids used in Figure 2—figure supplement 2, 3 and 4. [file elife-46259-supp1.docx]

| **PVC operon** | **Primer name** | **5’->3’ sequence** | **Product size** |
| --- | --- | --- | --- |
| *Pl*^TT01^Unit4 | TT01U4-BamHI_F | attggatcctcgctgttctctcttttcacc |  |
| *Pl*^TT01^Unit4 | TT01U4-KpnI_R | attggtacctggagttgtagacatgattttttcc | 499 |
| *Pl*^TT01^lopT | TT01LopT-BamHI_F | ataggatcccgctgtagtttgttttaaaaagg |  |
| *Pl*^TT01^lopT | TT01LopT-KpnI_R | attggtacctgtagttacggacatagtttatttcc | 499 |
| *Pl*^TT01^cif | TT01Cif-BamHI_F | ataggatcccgagcaataatgctgtgaat |  |
| *Pl*^TT01^cif | TT01Cif-KpnI_R | ataggtaccgacagttgtagacattgttatttcc | 499 |
| *Pl*^TT01^Unit1 | TT01U1-BamHI_F | ataggatccctactgggatgtgtattcaaaca |  |
| *Pl*^TT01^Unit1 | TT01U1-KpnI_R | attggtacctggagttatagccatgattcttc | 461 |
| *Pa*^PB68.1^Pnf | PB68 Pnf-BamHI_F | ataggatccatcccaacgtatcttgtcc |  |
| *Pa*^PB68.1^Pnf | PB68 Pnf-KpnI_R | attggtacctgtacttgtagacataaaagccc | 499 |
| *Pa*^PB68.1^lopT | PB68 LopT-BamHI_F | ataggatccccaataacctgacatattaaaccg |  |
| *Pa*^PB68.1^lopT | PB68 LopT-KpnI_R | attggtacctgtggttgtagtcataattatttcct | 499 |
| *Pa*^PB68.1^cif | PB68.1Cif-BamHI_F | ataggatccgcatgttattttcctgcctattat |  |
| *Pa*^PB68.1^cif | PB68.1Cif-KpnI_R | ataggtaccggcagttgtagacatcgtta | 543 |
| *Pa*^PB68.1^unit1 | PB68.1U1-BamHI_F | ataggatcccaattttaactatttactggacttcg |  |
| *Pa*^PB68.1^unit1 | PB68.1U1-KpnI_R | attggtacctggagttgtagacataatgtttcc | 486 |
